# Synthesis and Anti-HIV Activity of a Novel Series of Isoquinoline-Based CXCR4 Antagonists

**DOI:** 10.3390/molecules26206297

**Published:** 2021-10-18

**Authors:** Mastaneh Safarnejad Shad, Sandra Claes, Eline Goffin, Tom Van Loy, Dominique Schols, Steven De Jonghe, Wim Dehaen

**Affiliations:** 1 Department of Chemistry, Molecular Design and Synthesis, KU Leuven, Celestijnenlaan 200F, P.O. Box 2404, 3001 Leuven, Belgium; mastaneh.safarnejadshad@kuleuven.be (M.S.S.); eline.goffin@kuleuven.be (E.G.); 2 Laboratory of Virology and Chemotherapy, Department of Microbiology, Immunology and Transplantation, Rega Institute for Medical Research, KU Leuven, Herestraat 49, P.O. Box 1043, 3000 Leuven, Belgium; sandra.claes@kuleuven.be (S.C.); tom.vanloy@kuleuven.be (T.V.L.); dominique.schols@kuleuven.be (D.S.)

**Keywords:** isoquinoline, CXCR4 antagonist, HIV

## Abstract

An expansion of the structure–activity relationship study of CXCR4 antagonists led to the synthesis of a series of isoquinolines, bearing a tetrahydroquinoline or a 3-methylpyridinyl moiety as head group. All compounds were investigated for CXCR4 affinity and antagonism in competition binding and calcium mobilization assays, respectively. In addition, the anti-HIV activity of all analogues was determined. All compounds showed excellent activity, with compound **24c** being the most promising one, since it displayed consistently low nanomolar activity in the various assays.

## 1. Introduction

The CXC chemokine receptor 4 (CXCR4) is a seven transmembrane G protein-coupled receptor (GPCR), whose only endogenous ligand is the CXC chemokine ligand 12 (CXCL12), also known as stromal cell-derived factor 1 (SDF-1). Binding of CXCL12 to CXCR4 activates different signaling pathways, leading to various biological responses such as chemotaxis, cell survival and proliferation, intracellular calcium flux and gene transcription. Under normal physiological conditions, binding of CXCL12 induces leukocytes to migrate along chemokine gradients toward sites with high concentrations of CXCL12 [1]. Aberant CXCL12/CXCR4 signaling plays an important role in various pathological processes. Initially, CXCR4 was discovered as a coreceptor, in conjunction with the host CD4 receptor, for the entry of the T-tropic human immunodeficiency virus (HIV) into T-lymphocytes. CXCR4 is the main coreceptor of HIV-1 in the later stages of infection, that leads to a decrease in CD4 cell count and is linked to a higher chance of advancing to the acquired immune deficiency syndrome (AIDS) [2].

Mutations in the gene encoding for CXCR4 lead to truncation of its *C*-terminal tail and enhanced receptor activity. Clinically, it causes a rare combined immunodeficiency, characterized by warts, hypogammaglobulinemia, recurrent bacterial infection, and myelokathexis, which is known as the WHIM syndrome [3]. Overexpression of CXCR4 is reported for more than 20 types of solid and hematological cancers and is correlated with poor prognosis. CXCR4 was shown to be essential for various fundamental aspects of cancer, such as primary tumor growth, cancer cell migration, and the establishment of metastatic sites. This points toward CXCR4 as a general driver of human malignancies [4]. CXCR4 has also been implicated in various inflammatory disorders, such as rheumatoid arthritis, inflammatory bowel disease, and asthma [5].

**Figure 1:**
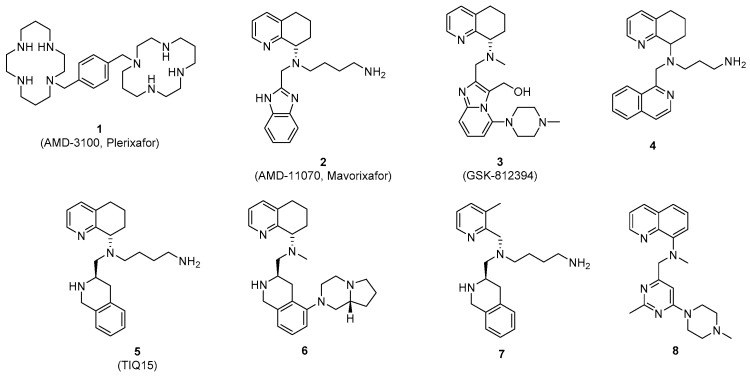
Small molecule CXCR4 antagonists.

The promise of CXCR4 as drug target spurred the search for small molecule CXCR4 antagonists [6,7]. AMD3100 (Plerixafor, compound **1**, Figure 1) received marketing approval for hematopoietic stem cell mobilization for transplantations in case of non-Hodgkin lymphoma and multiple myeloma [8]. In addition, AMD3100 has been used as a chemical tool to demonstrate that small molecule antagonism of CXCR4 is a promising strategy for the treatment of different cancers, such as breast cancer [9], prostate cancer [10], and ovarian cancer [11]. The lack of oral bioavailability of AMD3100 prompted the search for orally bioavailable small molecules. Different scaffolds have been elaborated, but most studies dealt with the tetrahydroquinoline-based derivatives. Among this class of compounds, AMD11070 (mavorixafor, compound **2**, Figure 1) is noteworthy. This analogue has been clinically evaluated as an antiretroviral agent, but was then halted for further development as an anti-HIV drug [12]. Recently, a phase 3 clinical trial for the treatment of the WHIM syndrome was initiated. Since then, many structural modifications have been pursued within this series. In most of these efforts, the 8-aminotetrahydroquinoline head piece was kept intact and structural variation was pursued on the lower part of the molecule. The basic *n*-butylamine side chain was removed and a piperazine ring was appended to the lower part, giving rise to GSK-812397 (compound **3**, Figure 1) [13]. It has been demonstrated that the benzimidazole moiety can be replaced by various mono- and bicyclic heteroaromatics, as exemplified by the isoquinoline congener **4** [14]. Substitution of the bicyclic heteroaromatic moiety by a partially saturated ring system yielded the tetrahydroisoquinoline derivative **5** (also known as TIQ15, Figure 1), which is a highly potent and selective CXCR4 antagonist [15]. Further optimization yielded compound **6**, which has improved in vitro ADME properties compared to compound **5** [16]. In contrast to the lower part, the upper tetrahydroquinoline moiety of AMD-11070 has been left relatively unexplored. It was shown that the stereochemistry of the tetrahydroquinoline moiety plays an important role in CXCR4 antagonism, with the (*S*)-enantiomer being more active than the (*R*)-enantiomer [17]. Structural simplification of the tetrahydroquinoline moiety afforded a series of 2-(aminomethyl)pyridine analogues, from which compound **7** (Figure 1) is a representative example. This compound displayed excellent CXCR4 antagonism and showed an improved metabolic stability (when compared to the corresponding tetrahydroquinoline congeners) [18]. Recently, it was shown that aromatization of the tetrahydroquinoline core yielded the aminoquinoline derivative **8** that displayed very potent CXCR4 affinity [19]. A major advantage of the open scaffolds and the fully aromatic compounds is the absence of chirality.

**Figure 2:**
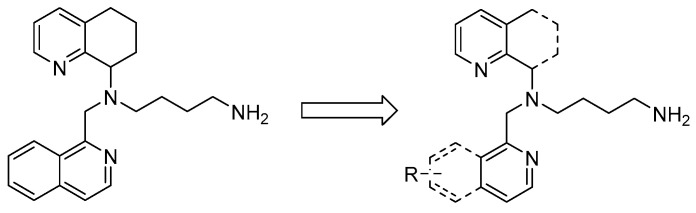
Newly designed CXCR4 antagonists.

In 2016, our group reported a metal-free three-component reaction for the synthesis of 1,5-disubstituted 1,2,3-triazoles [20,21,22], known as the “triazolization reaction of ketones”. In 2020, it was shown by our laboratory that the acid-mediated denitrogenative ring opening of triazoloisoquinolines furnished various 1-methyleneisoquinolines [23]. In this manuscript, these synthetic methodologies were applied for the synthesis of a series of novel isoquinoline-based CXCR4 antagonists, derived from the lead compound **4**. To probe the optimal substitution pattern for CXCR4 antagonism, various small substituents (R = F, Br, OCH_3_) were introduced on the isoquinoline moiety. In order to delineate if CXCR4 antagonism depends on the presence of a bicyclic isoquinoline moiety, the corresponding pyridine congeners were also prepared. As head groups, the classical 8-aminotetrahydroquinoline moiety, as well as the simplified 3-methylpyridinyl group, were selected (Figure 2).

## 2. Results and Discussion

### 2.1. Chemistry

**Scheme 1:**
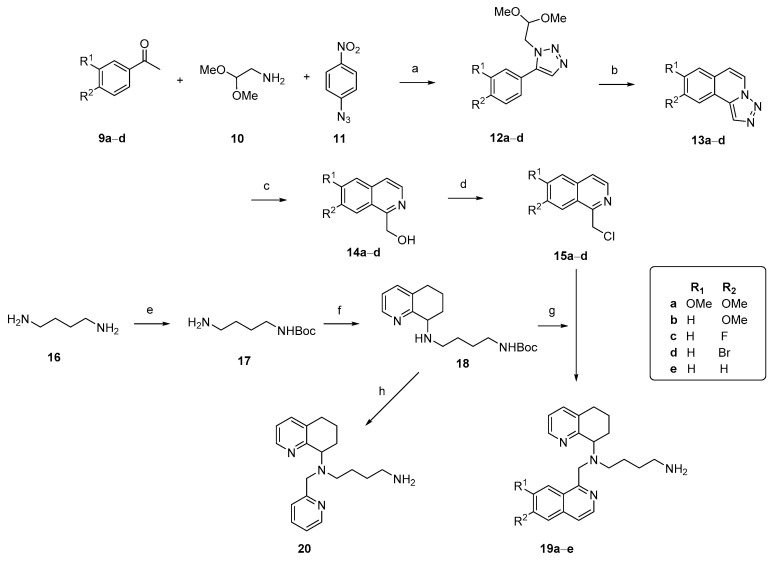
Reagents and conditions: (a) toluene, 100 °C, 17 h (62–89% yield); (b) H_2_SO_4_ (80–99 wt%), rt, 1–2 d (87–99% yield); (c) 2.5 M H_2_SO_4_, 120 °C, 1–2 d (76–92% yield); (d) SOCl_2_, DCM, rt, 3 h (90–99% yield); (e) Boc_2_O, DCM, rt, 17 h (65% yield); (f) 6,7-dihydroquinolin-8(5H)-one, NaBH(OAc)_3_, rt, 48 h (64% yield); (g): (i) K_2_CO_3_, MeCN, reflux, 2 d (61–86% yield); (ii) TFA, DCM, rt, 1 h (92–99% yield); (h) (i) picolinaldehyde, NaBH(OAc)_3_, DCE, rt, 2 d (63% yield); (ii) TFA, DCM, rt, 1 h (99% yield).

The synthesis of the tetrahydroquinoline derivatives is shown in Scheme 1. A multi-component reaction between acetophenone derivatives **9a**–**d**, 2,2-dimethoxyethan-1-amine **10** and 4-nitrophenyl azide (4-NPA) **11** in toluene at 100 °C yielded the dimethyl acetal-substituted triazoles **12a**–**d**. Conversion into the triazolo[5,1-*a*]isoquinolines **13a**–**d** was achieved using concentrated sulfuric acid in a modified Pomeranz-Fritsch reaction [23]. This was followed by an acid-catalyzed ring opening of the triazole moiety [24] using water as the nucleophile to afford the 1-hydroxymethylisoquinolines **14a**–**d**. Upon treatment with SOCl_2_ at room temperature, the desired 1-chloromethyl isoquinoline derivatives **15a**–**d** were obtained.

The selective mono-protection [25,26] of butane-1,4-diamine **16** with di-*tert*-butyl dicarbonate afforded intermediate **17**, which was used for further reaction without any purification. Reductive amination with 6,7-dihydroquinolin-8(5H)-one in the presence of NaBH(OAc)_3_ at room temperature, yielded compound **18** in 73% yield. The subsequent alkylation of amine **18** by treatment with 1-chloromethylisoquinolines **15a**–**e** in the presence of K_2_CO_3_ under reflux temperature was followed by acidic cleavage of the Boc protecting group, yielding final compounds **19a**–**e**. A reductive amination between amine **18** and the commercially available picolinaldehyde in presence of NaBH(OAc)_3_, followed by Boc deprotection, furnished the final compound **20** [14,27].

**Scheme 2:**
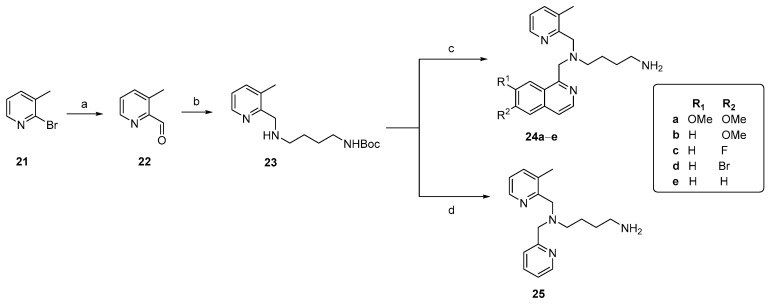
Reagents and conditions: (a) *n*BuLi (1.6 M in hexane), DMF, Et_2_O, 24 h (62% yield); (b) **17**, NaBH(OAc)_3_, DCE, rt, 48 h (74% yield); (c): (i) **15a**–**d**, K_2_CO_3_, MeCN, reflux, 2 d (57–84% yield); (ii) TFA, DCM, rt, 1 h (99% yield); (d): (i) picolinaldehyde, NaBH(OAc)_3_, DCE, rt, 2 d (52% yield); (ii) TFA, DCM, rt, 1 h (81% yield).

The synthesis of the 3-methylpyridine derivatives is depicted in Scheme 2. Formylation of the lithium salt of 2-bromo-3-methylpyridine **21** with DMF afforded 3-methylpicolinaldehyde **22** [28], which was then used in a reductive amination with amine **17**, yielding the key intermediate **23**. Alkylation of **23** with 1-chloromethylisoquinolines **15a**–**e** or a reductive amination with picolinaldehyde yielded target compounds **24a**–**e** and **25**, respectively [27].

### 2.2. Biological Evaluation

Compounds **19a**–**e**, **20**, **24a**–**e**, and **25** were evaluated in a panel of in vitro cell-based assays (Table 1) [29]. First, the ability of the compounds to compete with fluorescently labeled CXCL12 (CXCL12^AF647^) for binding at CXCR4 was determined. Second, since binding of CXCL12 to CXCR4 results into a transient increase of cytosolic calcium levels, the inhibition of this CXCL12-induced calcium mobilization by the various compounds was also investigated. Finally, given the role of CXCR4 as a major coreceptor for HIV entry, the antiviral activity of the compounds against the X4-tropic wild type HIV-1 NL4–3 strain and the HIV-2 ROD strain was determined in MT-4 cells. In parallel, the cytotoxicity of the compounds for uninfected MT-4 cells was assessed. In each of these assays, plerixafor was included as positive control and showed potent inhibitory activity in the various assays.

All newly synthesized isoquinoline-based derivatives were able to compete with CXCL12 for binding to CXCR4, as evidenced by IC_50_ values of less than 40 nM for all analogues, the only exception being compound **24b** that displayed an IC_50_ value of 0.89 µM in the binding assay. It is noteworthy that a structurally simplified analogue **25** carrying a 3-methylpyridinyl moiety (instead of the THQ group) and a pyridinyl ring (instead of the isoquinoline ring) is still endowed with very potent CXCR4-binding affinity (IC_50_ = 0.6 nM), making this a very ligand-efficient molecule. A similar trend was found in the CXCR4 calcium mobilization assay: all compounds were active (IC_50_ values of less than 1 µM), with compound **24b** being the least active (IC_50_ = 3.85 µM). However, all compounds do show a diminished potency in the calcium flux assay, when compared to the binding assay, which is also observed for plerixafor. The HIV screening revealed a number of analogues (compounds **19a**–**c**, **19e**, **20**, **24a**, and **24c**) that show excellent antiviral activity against HIV-1 and HIV-2 (EC_50_ < 100 nM) and at the same time, lack cytotoxicity for the MT4 cell line. Compounds that were less active in the calcium mobilization assay (such as compounds **19b**, **24b**, *24d*–**e**, and **25**) also displayed a diminished activity as anti-HIV agent. Overall, the biological profile of compound **24c** looks very promising, since it shows low nM activity in all assays (IC_50_ values between 0.6 and 6 nM) and lacks cytotoxicity (CC_50_ = 31 µM).

## 3. Materials and Methods

### 3.1. General Information

All chemicals were purchased from Acros Organics, Merck, Alfa Aesar, Fluorochem or TCI Europe and were used as received. For column chromatography, 70–230 mesh silica 60 (Acros) was used as the stationary phase. NMR spectra were recorded on commercial instruments (Bruker Avance 300 or Bruker Avance III HD 400 or Bruker Avance II+ 600) and chemical shifts (δ) are reported in parts per million (ppm) referenced to tetramethylsilane (^1^H), or the internal (NMR) solvent signal (^13^C). High-resolution mass spectra were acquired on a quadrupole orthogonal acceleration time-of-flight mass spectrometer (Synapt G2 HDMS, Waters, Milford, MA, USA). Samples were infused at 3 µL/min and spectra were obtained in positive ionization mode with a resolution of 15,000 (FWHM) using leucine enkephalin as lock mass. Melting points were determined using a Reichert Thermovar apparatus. Recombinant human CXCL12 was purchased from Peprotech and human CXCL12-Alexa 647^®^ (CXCL12^AF647^) was obtained from Almac.

### 3.2. Chemistry

#### 3.2.1. Synthesis of Dimethyl Acetal-Substituted Triazoles (**12a**–**d**)

**General procedure**

Commercially available substituted acetophenones **9a**–**d** (1 eq., 2.5 mmol) and 2-aminoacetaldehyde dimethyl acetal 10 (1.1 eq., 2.75 mmol, 289 mg) were added to an oven-dried screw-capped reaction tube equipped with a magnetic stirring bar. The mixture was dissolved in dry toluene (2 mL) followed by addition of 4-nitrophenyl azide 11 (1.5 eq., 3.75 mmol, 615 mg) and stirred overnight at 100 °C in an aluminum heating block. The crude residue was purified via silica gel flash chromatography, yielding the title compounds. The following compounds were made according to this general procedure.

- **1-(2,2-Dimethoxyethyl)-5-(3,4-dimethoxyphenyl)-1H-1,2,3-triazole (12a)**

This compound was prepared from 3′,4′-dimethoxyacetophenone **9a** (415.45 mg, 2.50 mmol). The crude residue was purified by silica gel flash chromatography using DCM/EtOAc (95:5) as mobile phase, yielding the title compound as a yellowish-orange solid (654 mg, 89%); mp 90–92 °C. **^1^H NMR** (400 MHz, CDCl_3_) δ: 7.67 (s, 1H), 7.10 (d, *J* = 2.0 Hz, 1H), 7.06 (dd, *J* = 8.2, 2.0 Hz, 1H), 6.97 (d, *J* = 8.2 Hz, 1H), 4.93 (t, *J* = 5.6 Hz, 1H), 4.41 (d, *J* = 5.7 Hz, 2H), 3.94 (s, 3H), 3.92 (s, 3H), 3.37 (s, 6H). **^13^C NMR** (101 MHz, CDCl3) δ: 150.0, 149.3, 139.0, 132.6, 122.1, 119.2, 112.4, 111.5, 103.7, 56.1, 56.1, 55.4, 49.7. HRMS (ESI-Q-TOF): *m*/*z* [M + H]^+^ calcd for C_14_H_19_N_3_O_4_: 294.1448; found: 294.1455.

- **1-(2,2-Dimethoxyethyl)-5-(4-methoxyphenyl)-1H-1,2,3-triazole (12b)**

This compound was prepared from 4′-methoxyacetophenone **9b** (375.45 mg, 2.50 mmol). The crude residue was purified by silica gel flash chromatography using DCM/EtOAc (95:5) as mobile phase, yielding the title compound as a yellowish-orange viscous oil (488 mg, 74%). **^1^H NMR** (400 MHz, CDCl_3_) δ: 7.64 (s, 1H), 7.42 (d, *J* = 8.7 Hz, 2H), 7.01 (d, *J* = 8.7 Hz, 2H), 4.88 (t, *J* = 5.6 Hz, 1H), 4.39 (d, *J* = 5.7 Hz, 2H), 3.85 (s, 3H), 3.33 (s, 6H). **^13^C NMR** (101 MHz, CDCl_3_) δ: 160.4, 138.7, 132.5, 130.5, 118.9, 114.4, 103.3, 55.3, 55.1, 49.4. HRMS (ESI-Q-TOF): *m*/*z* [M + H]^+^ calcd for C_13_H_17_N_3_O_3_: 264.1343; found: 264.1346.

- **1-(2,2-Dimethoxyethyl)-5-(4-fluorophenyl)-1H-1,2,3-triazole (12c)**

This compound was prepared from 4′-fluoroacetophenone **9c** (345.35 mg, 2.50 mmol). The crude residue was purified by silica gel flash chromatography using DCM/EtOAc (95:5) as the mobile phase, yielding the title compound as a reddish viscous oil (447 mg, 71%). **^1^H NMR** (400 MHz, CDCl_3_) δ: 7.65 (s, 1H), 7.47 (dd, *J* = 8.8, 5.2 Hz, 2H), 7.16 (t, *J* = 8.6 Hz, 2H), 4.86 (t, *J* = 5.6 Hz, 1H), 4.35 (d, *J* = 5.6 Hz, 2H), 3.33 (s, 6H). **^13^C NMR** (101 MHz, CDCl_3_) δ: 164.6, 162.1, 138.0, 132.9, 131.2, 122.9, 116.2, 103.6, 55.5, 49.6. HRMS (ESI-Q-TOF): *m*/*z* [M + H]^+^ calcd for C_12_H_14_FN_3_O_2_: 252.1143; found: 252.1139.

- **5-(4-Bromophenyl)-1-(2,2-dimethoxyethyl)-1H-1,2,3-triazole (12d)**

This compound was prepared from 4′-bromoacetophenone **9d** (497.6 mg, 2.50 mmol). The crude residue was purified by silica gel flash chromatography using DCM/EtOAc (95:5) as mobile phase, yielding the title compound as an orange viscous oil (485 mg, 62%). **^1^H NMR** (400 MHz, CDCl_3_) δ: 7.68 (s, 1H), 7.62 (d, *J* = 8.5 Hz, 2H), 7.37 (d, *J* = 8.5 Hz, 2H), 4.87 (t, *J* = 5.6 Hz, 1H), 4.36 (d, *J* = 5.6 Hz, 2H), 3.34 (s, 6H). **^13^C NMR** (101 MHz, CDCl_3_) δ: 138.1, 133, 132.4, 130.9, 125.9, 124.2, 103.7, 55.6, 49.8. HRMS (ESI-Q-TOF): *m*/*z* [M + H]^+^ calcd for C_12_H_14_BrN_3_O_2_: 312.0343; found: 312.0338.

#### 3.2.2. Synthesis of 1,2,3-Triazolo[5,1-a]isoquinolines (**13a**–**d**)

**General procedure**

To an open round-bottom flask equipped with a magnetic stirring bar containing the triazole **12a**–**d** (1.2 mmol) was added concentrated H_2_SO_4_ (80–95%, 2.5–4 mL), while stirring at 0 °C in an ice bath. After cooling for 30 min, the reaction mixture was stirred overnight at room temperature. The reaction mixture was poured over ice and neutralized by slowly adding a 3 M aqueous NaOH solution. The aqueous phase was subsequently extracted five times with dichloromethane. The combined organic layers were dried over MgSO_4_ and concentrated in vacuo. The crude residue was purified by flash chromatography, yielding the title compound. The following compounds were made according to this procedure.

- **8,9-Dimethoxy-[1,2,3]triazolo[5,1-a]isoquinoline (13a)**

This compound was prepared from triazole **12a** (354 mg, 1.21 mmol). The crude residue was purified by silica gel flash chromatography using DCM/EtOAc (80:20) as the mobile phase, yielding the title compound as an off-white solid (274 mg, 99%). mp 221–224 °C. **^1^H NMR** (400 MHz, CDCl_3_) δ: 8.42 (d, *J* = 7.3 Hz, 1H), 8.29 (s, 1H), 7.39 (s, 1H), 7.13 (s, 1H), 7.07 (d, *J* = 7.3 Hz, 1H), 4.05 (s, 3H), 4.02 (s, 3H). **^13^C NMR** (101 MHz, CDCl_3_) δ: 150.7, 150.6, 132.3, 124.5, 123.9, 121.0, 117.3, 115.4, 107.9, 104.5, 56.3, 56.2. HRMS (ESI-Q-TOF): *m*/*z* [M + H]^+^ calcd for C_12_H_11_N_3_O_2_: 230.0924; found: 230.0936.

- **8-Methoxy-[1,2,3]triazolo[5,1-*a*]isoquinoline (13b)**

This compound was prepared from triazole **12b** (301 mg, 1.14 mmol). The crude residue was purified by silica gel flash chromatography using DCM/EtOAc (80:20) as the mobile phase, yielding the title compound as an off-white solid (226 mg, 99%). mp 133–134 °C. **^1^H NMR** (400 MHz, CDCl_3_) δ: 8.43 (d, *J* = 7.4 Hz, 1H), 8.26 (s, 1H), 7.96 (d, *J* = 8.8 Hz, 1H), 7.20 (dd, *J* = 8.8, 2.6 Hz, 1H), 7.12 (d, *J* = 2.5 Hz, 1H), 7.06 (d, *J* = 7.4 Hz, 1H), 3.92 (s, 3H). **^13^C NMR** (101 MHz, CDCl_3_) δ: 160.0, 132.5, 130.7, 125.5, 124.6, 122.8, 118.3, 116.7, 115.6, 108.8, 55.5. HRMS (ESI-Q-TOF): *m*/*z* [M + H]^+^ calcd for C_11_H_9_N_3_O: 200.0818; found: 200.0826.

- **8-Fluoro-[1,2,3]triazolo[5,1-*a*]isoquinoline (13c)**

This compound was prepared from triazole **12c** (301 mg, 1.2 mmol) and H_2_SO_4_ (80%, aq.). The crude residue was purified by silica gel flash chromatography using DCM/EtOAc (80:20) as the mobile phase, yielding the title compound as an off-white solid (196 mg, 87%). mp 123–126 °C. **^1^H NMR** (400 MHz, CDCl_3_) δ: 8.55 (d, *J* = 7.4 Hz, 1H), 8.38 (s, 1H), 8.15 (dd, *J* = 8.6, 5.3 Hz, 1H), 7.47 (d, *J* = 9 Hz, 1H), 7.4 (d, *J* = 8.4 Hz, 1H), 7.15 (d, *J* = 7.4 Hz, 1H). **^13^C NMR** (101 MHz, CDCl_3_) δ 162.71 (d, *J* = 250.3 Hz), 138.48, 133.55, 130.96 (d, *J* = 8.5 Hz), 126.62 (d, *J* = 9.1 Hz), 125.63, 123.90, 117.79 (d, *J* = 23.9 Hz), 115.42 (d, *J* = 3.4 Hz), 113.05 (d, *J* = 22.2 Hz). HRMS (ESI-Q-TOF): *m*/*z* [M + H]^+^ calcd for C_10_H_6_FN_3_: 188.0618; found: 188.0617.

- **8-Bromo-[1,2,3]triazolo[5,1-*a*]isoquinoline (13d)**

This compound was prepared from triazole **12d** (375 mg, 1.2 mmol). The crude residue was purified by silica gel flash chromatography using DCM/EtOAc (80:20) as the mobile phase, yielding the title compound as an off-white solid (285 mg, 95%). mp 209–211 °C. **^1^H NMR** (400 MHz, CDCl_3_) δ: 8.54 (d, *J* = 7.4 Hz, 1H), 8.42 (s, 1H), 8.02 (d, *J* = 8.5 Hz, 1H), 7.97 (d, *J* = 1.9 Hz, 1H), 7.78 (dd, *J* = 8.5, 1.9 Hz, 1H), 7.3 (d, *J* = 7.4 Hz, 1H). **^13^C NMR** (101 MHz, CDCl_3_) δ: 131.8, 131.7, 130.3, 129.8, 125.7, 125.3, 123.5, 1229, 121.4, 114.6. HRMS (ESI-Q-TOF): *m*/*z* [M + H]^+^ calcd for C_10_H_6_BrN_3_: 247.9818; found: 247.9812.

#### 3.2.3. Synthesis of 1-Hydroxymethylisoquinolines (**14a**–**d**)

**General procedure**

A solution of the triazoloisoquinoline **13a**–**d** (1.2 mmol) in a 2.5M H_2_SO_4_ solution (8–10 mL) was heated at 120 °C until completion of the reaction (24–36 h). Subsequently, the reaction mixture was poured over ice and neutralized by adding a saturated aqueous NaHCO_3_ solution. The aqueous phase was extracted three times with dichloromethane. The combined organic phases were dried over MgSO_4_ and concentrated in vacuo to afford the title compound, which was used for the next step without further purification. The following compounds were made according to this procedure.

- **(6,7-Dimethoxyisoquinolin-1-yl)methanol (14a)**

This compound was prepared from triazoloisoquinoline **13a** (275 mg, 1.2 mmol). The title compound was isolated as an off-white solid (242 mg, 92%). mp 131–133 °C. **^1^H NMR** (400 MHz, CDCl_3_) δ: 8.33 (d, *J* = 5.7, 1H), 7.46 (d, *J* = 5.6, 1H), 7.11 (s, 1H), 7.05 (s, 1H), 5.13 (s, 2H), 4.04 (s, 3H), 4.03 (s, 3H). **^13^C NMR** (101 MHz, CDCl_3_) δ: 155.5, 153.7, 151.1, 140.1, 133.5, 119.9, 106.1, 101.9, 62.1, 56.8. HRMS (ESI-Q-TOF): *m*/*z* [M + H]^+^ calcd for C_12_H_13_NO_3_: 220.0968; found: 220.0974.

- **(6-Methoxyisoquinolin-1-yl)methanol (14b)**

This compound was prepared from triazoloisoquinoline **13b** (240 mg, 1.2 mmol). The title compound was isolated as an off-white solid (173 mg, 76%); mp 63–65 °C. **^1^H NMR** (400 MHz, CDCl_3_) δ: 8.30 (d, *J* = 5.8 Hz, 1H), 7.76 (d, *J* = 9.2 Hz, 1H), 7.43 (d, *J* = 5.7 Hz, 1H), 7.19 (s, 1H), 7.04 (s, 1H), 5.11 (s, 2H), 3.88 (s, 3H). **^13^C NMR** (101 MHz, CDCl_3_) δ: 160.7, 156.5, 140.5, 137.9, 124.8, 120.2, 120.1, 119.5, 104.8, 60.9, 55.2. HRMS (ESI-Q-TOF): *m*/*z* [M + H]^+^ calcd for C_11_H_11_NO_2_: 190.0862; found: 190.0869.

- **(6-Fluoroisoquinolin-1-yl)methanol (14c)**

This compound was prepared from triazoloisoquinoline **13c** (225 mg, 1.2 mmol). The title compound was isolated as an off-white solid (194 mg, 91%). mp 72–74°C. **^1^H NMR** (400 MHz, CDCl_3_) δ: 8.45 (d, *J* = 5.8 Hz, 1H), 7.96 (dd, *J* = 9.1, 5.3 Hz, 1H), 7.57 (d, *J* = 5.8 Hz, 1H), 7.48 (dd, *J* = 9.2, 2.5 Hz, 1H), 7.3 (td, *J* = 8.5, 2.5 Hz, 1H), 5.22 (s, 2H), 4.93 (s, 1H). **^13^C NMR** (101 MHz, CDCl_3_) δ: 163.9, 161.4, 156.9, 140.8, 137.1, 125.8, 121.6, 119.4, 117.4, 110.3. HRMS (ESI-Q-TOF): *m*/*z* [M + H]^+^ calcd for C_10_H_8_FNO: 178.0663; found: 178.0659.

- **(6-Bromoisoquinolin-1-yl)methanol (14d)**

This compound was prepared from triazoloisoquinoline **13d** (298 mg, 1.2 mmol). The title compound was isolated as an off-white solid (238 mg, 83%). mp 143–145°C. **^1^H NMR** (400 MHz, CDCl_3_) δ: 8.47 (d, *J* = 5.4 Hz, 1H), 8.03 (d, *J* = 1.8 Hz, 1H), 7.79 (d, *J* = 8.9 Hz, 1H), 7.69 (dd, *J* = 8.9, 1.9 Hz, 1H), 7.51 (d, *J* = 5.7 Hz, 1H), 5.20 (s, 2H), 4.89 (s, 1H). **^13^C NMR** (101 MHz, CDCl_3_) δ: 158.2, 137.5, 131.6, 130, 125.7, 125.4, 123.8, 119.7, 61.8. HRMS (ESI-Q-TOF): *m*/*z* [M + H]^+^ calcd for C_10_H_8_BrNO: 237.9862; found: 237.9851

#### 3.2.4. Synthesis of 1-(Chloromethyl)isoquinolines (**15a**–**d**)

**General procedure**

To a solution of 1-hydroxymethylisoquinoline **14a**–**d** (1.2 mmol) in dry dichloromethane (1 mL) was added an excess of SOCl_2_. The resulting reaction mixture was stirred for 3 h at room temperature. After completion of the reaction, the reaction mixture was neutralized with an aqueous saturated NaHCO_3_ solution. The aqueous phase was extracted several times with dichloromethane. The combined organic layers were dried over MgSO_4_ and concentrated under vacuum to afford the title compound, that was used for the next step without further purification. The following compounds were made according to this procedure.

- **1-(Chloromethyl)-6,7-dimethoxyisoquinoline (15a)**

This compound was prepared from 1-hydroxymethylisoquinoline **14a** (263 mg, 1.2 mmol). The title compound was isolated as a red-orange solid; yield: 285 mg (99%); mp 148–150 °C. **^1^H NMR** (400 MHz, CDCl_3_) δ: 8.34 (d, *J* = 5.8 Hz, 1H), 7.50 (d, *J* = 5.5 Hz, 1H), 7.42 (s, 1H), 7.09 (s, 1H), 5.09 (s, 2H), 4.07 (s, 3H), 4.03 (s, 3H). **^13^C NMR** (101 MHz, CDCl_3_) δ: 153.1, 153.0, 150.6, 141.0, 133.8, 122.7, 120.7, 105.4, 103.2, 56.2, 56.2, 45.7. HRMS (ESI-Q-TOF): *m*/*z* [M + H]^+^ calcd for C_12_H_12_ClNO_2_: 238.0629; found: 238.0631.

- **1-(Chloromethyl)-6-methoxyisoquinoline (15b)**

This compound was prepared from 1-hydroxymethylisoquinoline **14b** (227 mg, 1.2 mmol). The title compound was isolated as a dark red solid (225 mg, 90%). mp 85–87 °C. **^1^H NMR** (400 MHz, CDCl_3_) δ: 8.40 (d, *J* = 5.7 Hz, 1H), 8.15 (d, *J* = 9.2 Hz, 1H), 7.55 (d, *J* = 5.7 Hz, 1H), 7.29 (dd, *J* = 9.2, 2.5 Hz, 1H), 7.10 (d, *J* = 2.5 Hz, 1H), 5.09 (s, 2H), 3.96 (s, 3H). **^13^C NMR** (101 MHz, CDCl_3_) δ: 160.9, 154.7, 141.7, 138.9, 126.8, 121.0, 120.8, 104.8, 55.4, 44.3. HRMS (ESI-Q-TOF): *m*/*z* [M + H]^+^ calcd for C_11_H_10_ClNO: 208.0524; found: 208.0516.

- **1-(Chloromethyl)-6-fluoroisoquinoline (15c)**

This compound was prepared from 1-hydroxymethylisoquinoline **14c** (213 mg, 1.2 mmol). The title compound was isolated as a red solid (224 mg, 95%). mp 65–67 °C. **^1^H NMR** (600 MHz, CDCl_3_) δ: 8.47 (d, *J* = 5.7 Hz, 1H), 8.30 (dd, *J* = 9.1, 5.3 Hz, 1H), 7.62 (d, *J* = 5.7 Hz, 1H), 7.52–7.39 (m, 2H), 5.12 (s, 2H). **^13^C NMR** (151 MHz, CDCl_3_) δ: 164, 162.4, 155.9, 142.9, 138.6, 128.5, 123.8, 121.6, 118.5, 110.9, 45. HRMS (ESI-Q-TOF): *m*/*z* [M + H]^+^ calcd for C_10_H_7_ClFN: 196.0324; found: 196.0316.

- **6-Bromo-1-(chloromethyl)isoquinoline (15d)**

This compound was prepared from 1-hydroxymethylisoquinoline **14d** (286 mg, 1.2 mmol). The title compound was isolated as a reddish solid (301 mg, 98%). mp 108–110 °C. **^1^H NMR** (400 MHz, CDCl_3_) δ: 8.50 (d, *J* = 5.7 Hz, 1H), 8.14 (d, *J* = 9 Hz, 1H), 8.05 (d, *J* = 1.9 Hz, 1H), 7.75 (dd, *J* = 9, 1.9 Hz, 1H), 7.58 (d, *J* = 7.6 Hz, 1H), 5.12 (s, 2H). **^13^C NMR** (101 MHz, CDCl_3_) δ: 156.1, 143.1, 137.9, 131.5, 129.8, 127, 125.4, 125, 120.8, 44.8. HRMS (ESI-Q-TOF): *m*/*z* [M + H]^+^ calcd for C_10_H_7_BrClN: 255.9524; found: 255.9520.

#### 3.2.5. *tert*-Butyl (4-aminobutyl) carbamate (**17**)

To a solution of 1,4-diaminobutane **16** (10 eq., 56.7 mmol, 5 g) in dichloromethane (62.5 mL) was added a solution of Boc_2_O (1 eq., 5.67 mmol, 1.238 g) in dichloromethane (62.5 mL) through a dropping funnel. The reaction mixture was stirred at room temperature for 16 h. After filtering the suspension, the filtrate was evaporated under vacuum. The oily residue was washed with brine and extracted with EtOAc to remove the excess amount of diamine. The organic layer was dried over anhydrous Na_2_SO_4_ and evaporated under vacuum, yielding the title compound as a colorless oil (6.9 g, 65%). **^1^H NMR** (400 MHz, CDCl_3_) δ: 4.77 (s, 1H), 3.07 (t, *J* = 6.0 Hz, 2H), 2.66 (t, *J* = 6.6 Hz, 2H), 1.52–1.42 (m, 4H), 1.39 (s, 9H), 1.09 (s, 2H). **^13^C NMR** (75 MHz, CDCl_3_) δ: 155.88, 78.41, 41.51, 40.09, 30.59, 28.17, 27.19. HRMS (ESI-Q-TOF): *m*/*z* [M + H]^+^ calcd for C_9_H_20_N_2_O_2_: 189.1597; found: 189.1597.

#### 3.2.6. *tert*-Butyl (4-((5,6,7,8-tetrahydroquinolin-8-yl)amino)butyl) carbamate (**18**)

6,7-Dihydroquinolin-8(5H)-one (1.03 eq., 3.40 mmol, 0.500 g) was added to a slurry of NaBH(OAc)_3_ (1.78 eq., 5.87 mmol, 1.244 g) in dichloroethane (3 mL), followed by the addition of *tert*-butyl (4-aminobutyl) carbamate **17** (1 eq., 3.30 mmol, 0.621 g). The reaction was stirred at room temperature for 48 h. The reaction mixture was quenched with a 1N NaOH solution to obtain a pH ~ 8 of the aqueous layer. The reaction mixture was extracted three times with dichloromethane (3×) and the combined organic layers were concentrated to a volume of approximately 3 mL. Heptane (10 mL) was added and the volume was concentrated to 5 mL. Upon cooling the reaction mixture to room temperature, a precipitate was formed. After further cooling the suspension to 0 °C, the precipitate was filtered off and dried under vacuum, yielding the title compound as a light brown solid (674 mg, 64%); mp 73–75 °C. **^1^H NMR** (400 MHz, CDCl_3_) δ: 8.39 (d, *J* = 4.1 Hz, 1H), 7.82 (s, 1H), 7.41 (d, *J* = 7.8 Hz, 1H), 7.12 (t, *J* = 7.4, 4.9 Hz, 1H), 4.01 (s, 1H), 3.09 (t, *J* = 33.0 Hz, 2H), 2.90–2.70 (m, 4H), 2.32–2.00 (m, 2H), 1.95 (t, *J* = 16.3 Hz, 2H), 1.73–1.62 (m, 2H), 1.62–1.51 (m, 2H), 1.42 (s, 9H). **^13^C NMR** (101 MHz, CDCl_3_) δ: 156.31, 147.03, 137.27, 132.95, 122.48, 57.79, 46.09, 28.64, 27.77, 27.40, 26.52, 22.02, 20.18. HRMS (ESI-Q-TOF): *m*/*z* [M + H]^+^ calcd for C_18_H_29_N_3_O_2_: 320.2332; found: 320.2335. 

#### 3.2.7. Coupling of 1-(Chloromethyl)isoquinolines **15a**–**d** with Amine **18**

**General procedure**

To a solution of 1-(chloromethyl)isoquinoline **15a**–**d** (1.5 eq.) and *tert*-butyl (4-((5,6,7,8-tetrahydroquinolin-8-yl)amino)butyl)carbamate **18** (1 eq.) in dry acetonitrile (5 mL) was added K_2_CO_3_ (8 eq.). The reaction mixture was refluxed for 24–48 h. The mixture was cooled to room temperature and filtered through a Celite^®^ pad. The filtrate was evaporated in vacuo and the crude residue was purified via silica gel flash chromatography using a mixture of Et_2_O/MeOH as the mobile phase (in a gradient gradually ranging from 100:0 to 90:10) yielding the title compounds. The following compounds were made according to this procedure.

- ***tert*-Butyl(4-(((6,7-dimethoxyisoquinolin-1-yl)methyl)(5,6,7,8-tetrahydroquinolin-8 yl)amino)butyl) carbamate**

This compound was prepared from 1-(chloromethyl)-6,7-dimethoxyisoquinoline **15a** (86 mg, 0.36 mmol) and *tert*-butyl (4-((5,6,7,8-tetrahydroquinolin-8-yl)amino)butyl)carbamate **18** (100 mg, 0.32 mmol). The title compound was isolated as a brown viscous oil (114 mg, 69%). **^1^H NMR** (300 MHz, CDCl_3_) δ: 8.52 (s, 1H), 8.43 (d, *J* = 4.2 Hz, 1H), 8.26 (d, *J* = 5.6 Hz, 1H), 7.37 (d, *J* = 5.6 Hz, 1H), 7.30 (d, *J* = 7.5 Hz, 1H), 7.03–6.97 (m, 2H), 4.62 (s, 1H), 4.35 (dd, *J* = 56.5, 12.4 Hz, 2H), 4.14 (s, 3H), 4.00 (s, 3H), 2.97–2.46 (m, 6H), 2.12–1.90 (m, *J* = 22.3, 11.4 Hz, 3H), 1.70–1.54 (m, 1H), 1.39 (s, 9H), 1.34–1.16 (m, *J* = 28.5, 11.3 Hz, 4H).**^13^C NMR** (75 MHz, CDCl_3_) δ: 156.09, 152.72, 149.61, 147.10, 140.57, 136.49, 134.38, 133.31, 124.39, 121.55, 119.29, 106.62, 104.75, 77.36, 60.90, 58.81, 56.51, 55.99, 50.53, 29.55, 28.54, 27.74, 25.31, 24.94, 21.80. HRMS (ESI-Q-TOF): *m*/*z* [M + H]^+^ calcd for C_30_H_40_N_4_O_4_: 521.3122; found: 521.3124.

- ***tert*-Butyl(4-(((6-methoxyisoquinolin-1-yl)methyl)(5,6,7,8-tetrahydroquinolin-8-yl) amino)butyl) carbamate**

This compound was prepared from 1-(chloromethyl)-6-methoxyisoquinoline **15b** (104 mg, 0.33 mmol) and *tert*-butyl (4-((5,6,7,8-tetrahydroquinolin-8-yl)amino)butyl)carbamate **18** (101.4 mg, 0.49 mmol). The title compound was isolated as a brown viscous oil (136 mg, 85%). **^1^H NMR** (400 MHz, CDCl_3_) δ: 8.66 (d, *J* = 9.2 Hz, 1H), 8.54 (d, *J* = 3.9 Hz, 1H), 8.32 (d, *J* = 5.8 Hz, 1H), 7.43 (d, *J* = 5.8 Hz, 1H), 7.36 (d, *J* = 7.4 Hz, 1H), 7.18 (dd, *J* = 9.3, 2.5 Hz, 1H), 7.08 (dd, *J* = 7.6, 4.6 Hz, 1H), 7.01 (d, *J* = 2.6 Hz, 1H), 4.89 (s, 1H), 4.38 (s, 1H), 4.24 (s, 2H), 3.94 (s, 3H), 2.94–2.56 (m, 6H), 2.42–2.12 (m, 4H), 2.15–1.99 (m, 4H), 1.39 (s, 9H). **^13^C NMR** (101 MHz, CDCl_3_) δ: 161.5, 156.8, 147.8, 142.5, 139.1, 137.3, 135.2, 129.9, 122.5, 120.6, 120, 105, 56.1, 51.5, 40.5, 30.4, 30, 29.2, 28.2, 25.4, 22.2. HRMS (ESI-Q-TOF): *m*/*z* [M + H]^+^ calcd for C_29_H_38_N_4_O_3_: 491.3016; found: 491.3017.

- ***tert*-Butyl(4-(((6-fluoroisoquinolin-1-yl)methyl)(5,6,7,8-tetrahydroquinolin-8-yl) amino)butyl) carbamate**

This compound was prepared from 1-(chloromethyl)-6-fluoroisoquinoline **15c** (117 mg, 0.6 mmol) and *tert*-butyl (4-((5,6,7,8-tetrahydroquinolin-8-yl)amino)butyl)carbamate **18** (127.8 mg, 0.4 mmol). The title compound was isolated as a brown viscous oil (164 mg, 86%). **^1^H NMR** (400 MHz, CDCl_3_) δ: 8.95 (dd, *J* = 8.9, 5.8 Hz, 1H), 8.51 (d, *J* = 3.9 Hz, 1H), 8.36 (d, *J* = 5.7 Hz, 1H), 7.47 (d, *J* = 5.7 Hz, 1H), 7.38–7.29 (m, 3H), 7.05 (dd, *J* = 7.6, 4.7 Hz, 1H), 4.80 (s, 1H), 4.36 (d, *J* = 12.5 Hz, 1H), 4.20 (d, *J* = 3.6 Hz, 2H), 2.95–2.52 (m, 6H), 2.14–1.97 (m, 2H), 1.88–1.63 (m, 4H), 1.39 (s, 9H), 1.30–1.22 (m, 2H). **^13^C NMR** (101 MHz, CDCl_3_) δ: 155.7, 146.8, 141.9, 137.7, 137.6, 136.3, 134.2, 130.8, 124.9, 121.4, 119.8, 109.6, 109.3, 78.5, 60.8, 57.2, 53.2, 50.6, 39.7, 33.3, 29.4, 29, 28.1, 27.2, 24.5, 24, 21.2. HRMS (ESI-Q-TOF): *m*/*z* [M + H]^+^ calcd for C_28_H_35_FN_4_O_2_: 479.2817; found: 479.2808.

- ***tert*-Butyl(4-(((6-bromoisoquinolin-1-yl)methyl)(5,6,7,8-tetrahydroquinolin-8-yl) amino)butyl) carbamate**

This compound was prepared from 1-(chloromethyl)-6-bromoisoquinoline **15d** (142 mg, 0.55 mmol) and *tert*-butyl (4-((5,6,7,8-tetrahydroquinolin-8-yl)amino)butyl)carbamate **18** (117.9 mg, 0.37 mmol). The title compound was isolated as a brown viscous oil (143 mg, 72%).**^1^H NMR** (400 MHz, CDCl_3_) δ: 8.78 (d, *J* = 8.9 Hz, 1H), 8.53 (d, *J* = 3.8 Hz, 1H), 8.40 (d, *J* = 5.7 Hz, 1H), 7.92 (d, *J* = 1.9 Hz, 1H), 7.64 (dd, *J* = 9, 1.97 Hz, 1H), 7.43 (d, *J* = 5.7 Hz, 1H), 7.35 (d, *J* = 7.6 Hz, 1H), 7.06 (dd, *J* = 7.6, 4.69 Hz, 1H), 4.80 (s, 1H), 4.36 (d, *J* = 12, 1H), 4.20 (d, *J* = 10 Hz, 2H), 2.95–2.54 (m, 6H), 2.11–2.03 (m, 2H), 1.75–1.58 (m, 4H), 1.41 (s, 9H), 1.21 (t, *J* = 7 Hz, 2H). **^13^C NMR** (101 MHz, CDCl_3_) δ: 155.9, 146.8, 141.9, 137.6, 137.3, 136.6, 130.2, 128.9, 128.7, 124.8, 123.2, 121.8, 119.3, 61.2, 57.1, 50.8, 39.6, 30.7, 29.3, 28.9, 28.2, 27.2, 27.5, 26.9, 24.3, 24, 21.2, 19.9. HRMS (ESI-Q-TOF): *m*/*z* [M + H]^+^ calcd for C_28_H_35_BrN_4_O_2_: 539.2016; found: 539.2021.

- ***tert*-Butyl(4-((isoquinolin-1-ylmethyl)(5,6,7,8-tetrahydroquinolin-8-yl)amino)butyl) carbamate**

This compound was prepared from the commercially available 1-(bromomethyl)isoquinoline (200 mg, 0.9 mmol) and *tert*-butyl (4-((5,6,7,8-tetrahydroquinolin-8-yl)amino)butyl)carbamate **18** (191.78 mg, 0.6 mmol). The title compound was isolated as a brown viscous oil (169 mg, 61%). **^1^H NMR** (400 MHz, CDCl_3_) δ: 8.52 (d, *J* = 3.8 Hz, 1H), 8.09 (d, *J* = 8.5 Hz, 1H), 8.00 (d, *J* = 8.4 Hz, 1H), 7.94 (d, *J* = 8.5 Hz, 1H), 7.77 (d, *J* = 7.7 Hz, 1H), 7.68–7.61 (m, 1H), 7.52–7.43 (m, 1H), 7.33 (d, *J* = 7.5 Hz, 1H), 7.04 (dd, *J* = 7.6, 4.7 Hz, 1H), 4.86 (s, 1H), 4.21 (dd, *J* = 8.3, 6.6 Hz, 1H), 4.01–3.87 (m, 2H), 2.99 (s, 2H), 2.86–2.64 (m, 4H), 2.11–1.87 (m, 2H), 1.54–1.44 (m, 2H), 1.42 (s, 9H), 1.29–1.20 (m, 2H). **^13^C NMR** (101 MHz, CDCl_3_) δ: 156.5, 147.7, 137.1, 136.6, 134.8, 129.6, 129.2, 128, 127.9, 126.3, 122.1, 121.9, 61.5, 58.9, 53.9, 52.8, 40.7, 30.2, 29.7, 28.9, 28.1, 26, 21.9. HRMS (ESI-Q-TOF): *m*/*z* [M + H]^+^ calcd for C_28_H_36_N_4_O_2_: 461.2911; found: 461.2935.

#### 3.2.8. Synthesis of 3-Methylpicolinaldehyde (**22**)

To a flame-dried flask under an inert argon atmosphere was added 2-bromo-3-methyl-pyridine **21** (1 eq., 11.40 mmol, 2 g) and Et_2_O (1–2 mL) at −78°C, followed by the addition of a 1.6 M butyllithium solution in hexane (0.43 mL). After stirring for 3 h at −78 °C, DMF (2 mL) was added to this deep red-brown solution. The reaction temperature was gradually increased to room temperature and was stirred for an additional 22 h. The reaction mixture was quenched through the dropwise addition of ice-water and extracted with Et_2_O. The organic layer was washed with a 5% aqueous NaHCO_3_ solution and brine. The combined organic layers were dried over MgSO_4_ and concentrated under vacuum. The crude residue was purified by silica gel flash chromatography using Et_2_O/petroleum ether (80:20) as the eluent, yielding the title compound as an orange-red oil (848 mg, 62%).

#### 3.2.9. *tert*-Butyl(4-(((3-methylpyridin-2-yl)methyl)amino)butyl) carbamate (**23**)

3-Methylpicolinaldehyde **22** (1 eq., 8.25 mmol, 1 g) was added to a slurry of NaBH(OAc)_3_ (1.9 eq., 15.68 mmol, 3.324 g) in dichloroethane (15 mL), followed by the addition of *tert*-butyl (4-aminobutyl) carbamate **17** (1.4 eq., 11.56 mmol, 2.176 g). The reaction was stirred at room temperature for 48 h. The reaction mixture was quenched with a 1 N NaOH solution to obtain a pH ~ 8 in the aqueous layer. After extracting the mixture with dichloromethane (3×), the combined organic phases were concentrated to a volume of approximately 3 mL. The crude residue was purified by silica gel column chromatography using Et_2_O/MeOH (96:4) as the eluent, affording the title compound as a yellowish viscous oil (1.8 g, 74%). **^1^H NMR** (400 MHz, CDCl_3_) δ: 8.37 (d, *J* = 3.84 Hz, 1H), 7.41 (dd, *J* = 7.6, 0.64 Hz, 1H), 7.06 (dd, *J* = 7.5, 4.8 Hz, 1H), 4.79 (s, 1H), 3.86 (s, 2H), 3.17–3.06 (m, 2H), 2.94 (s, 1H), 2.72 (t, *J* = 6.7 Hz, 2H), 2.29 (s, 3H), 1.64–1.49 (m, 4H), 1.42 (s, 9H). **^13^C NMR** (101 MHz, CDCl_3_) δ: 157.1, 156.1, 146.5, 137.7, 130.9, 121.9, 79, 52, 49.5, 40.5, 28.5, 27.4, 18.1. HRMS (ESI-Q-TOF): *m*/*z* [M + H]^+^ calcd for C_16_H_27_N_3_O_2_: 294.2176; found: 294.2169.

#### 3.2.10. Coupling of 1-(Chloromethyl)isoquinolines **15a**–**d** with amine **23**

A similar procedure as mentioned earlier for the coupling with amine **18** was used. The following compounds were made according to this procedure.

- ***tert*-Butyl(4-(((6,7-dimethoxyisoquinolin-1-yl)methyl)((3-methylpyridin-2-yl)methyl)amino)butyl) carbamate**

This compound was prepared from 1-(chloromethyl)-6,7-dimethoxyisoquinoline **15a** (50 mg, 0.21 mmol) and *tert*-butyl (4-(((3-methylpyridin-2-yl)methyl)amino)butyl)carbamate **23** (48 mg, 0.15 mmol). The title compound was isolated as a brownish viscous oil (76 mg, 73%). **^1^H NMR** (400 MHz, CDCl_3_) δ: 8.38 (d, *J* = 4.1 Hz, 1H), 8.30 (d, *J* = 5.6 Hz, 1H), 7.51 (s, 1H), 7.42 (d, *J* = 5.6 Hz, 1H), 7.36 (d, *J* = 7.4 Hz, 1H), 7.08 (dd, *J* = 7.6, 4.8 Hz, 1H), 7.02 (s, 1H), 4.75 (s, 1H), 4.23 (s, 2H), 4.00 (s, 3H), 3.82 (s, 5H), 2.96–2.83 (m, 2H), 2.59 (s, 2H), 2.10 (s, 3H), 1.64–1.51 (m, 2H), 1.40 (s, 9H), 1.30 (t, *J* = 6.9 Hz, 2H). **^13^C NMR** (101 MHz, CDCl_3_) δ: 162.5, 156.8, 155.9, 151.9, 151.3, 147.2, 141.2, 136.8, 133.1, 131.9, 123.9, 121.3, 117.7, 105.4, 79.5, 67.6, 59.1, 56.1, 54, 35.8, 28.4, 27.7, 25.8, 20.4. HRMS (ESI-Q-TOF): *m*/*z* [M + H]^+^ calcd for C_28_H_38_N_4_O_4_: 495.2966; found: 495.2960.

- ***tert*-Butyl(4-(((6-methoxyisoquinolin-1-yl)methyl)((3-methylpyridin-2-yl)methyl) amino)butyl) carbamate**

This compound was prepared from 1-(chloromethyl)-6-methoxyisoquinoline **15b** (100 mg, 0.48 mmol) and *tert*-butyl (4-(((3-methylpyridin-2-yl)methyl)amino)butyl)carbamate **23** (94.2 mg, 0.32 mmol). The title compound was isolated as a brownish viscous oil (118 mg, 57%). **^1^H NMR** (400 MHz, CDCl_3_) δ: 8.38 (d, *J* = 3.8 Hz, 1H), 8.33 (d, *J* = 5.8 Hz, 1H), 7.88 (d, *J* = 9 Hz, 1H), 7.43 (d, *J* = 5.8 Hz, 1H), 7.40 (d, *J* = 7.1 Hz, 1H), 7.10 (dd, *J* = 7.5, 4.8 Hz, 1H), 7.06–6.98 (m, 2H), 4.71 (s, 1H), 4.14 (s, 2H), 3.91 (s, 3H), 3.83 (s, 2H), 2.94–2.82 (m, 2H), 2.57 (t, *J* = 7.2 Hz, 2H), 2.10 (s, 3H), 1.58–1.46 (m, 2H), 1.44 (s, 9H), 1.32–1.17 (m, 2H). **^13^C NMR** (101 MHz, CDCl_3_) δ: 160.2, 155.8, 146, 141.8, 138.2, 137.8, 133.2, 128.1, 123.2, 122.3, 119.7, 119.1, 104.1, 78.7, 59.1, 55.2, 53.7, 39.7, 28.2, 27.4, 22.7, 18. HRMS (ESI-Q-TOF): *m*/*z* [M + H]^+^ calcd for C_27_H_36_N_4_O_3_: 465.2860; found: 465.2863.

- ***tert*-Butyl(4-(((6-fluoroisoquinolin-1-yl)methyl)((3-methylpyridin-2-yl)methyl) amino)butyl) carbamate**

This compound was prepared from 1-(chloromethyl)-6-fluoroisoquinoline **15c** (120 mg, 0.61 mmol) and *tert*-butyl (4-(((3-methylpyridin-2-yl)methyl)amino)butyl)carbamate **23** (120 mg, 0.41 mmol). The title compound was isolated as a brown viscous oil (150 mg, 81%). **^1^H NMR** (400 MHz, CDCl_3_) δ: 8.41 (d, *J* = 5.7 Hz, 2H), 8.06 (dd, *J* = 9.2, 5.7 Hz, 1H), 7.49 (d, *J* = 5.8 Hz, 1H), 7.42 (d, *J* = 6.9 Hz, 1H), 7.35 (dd, *J* = 9.3, 2.5 Hz, 1H), 7.17 (dd, *J* = 8.8, 2.4 Hz, 1H), 7.12 (dd, *J* = 7.6, 4.9 Hz, 1H), 4.66 (s, 1H), 4.16 (s, 2H), 3.83 (s, 2H), 2.97–2.86 (m, 2H), 2.56 (t, *J* = 7.3 Hz, 2H), 2.13 (s, 3H), 1.59–1.48 (m, 2H), 1.42 (s, 9H), 1.31–1.19 (m, 2H). **^13^C NMR** (101 MHz, CDCl_3_) δ: 164.1, 161.6, 159, 156.9, 156.1, 146.4, 142.4, 138, 133.3, 129.9, 125, 122.6, 120.2, 116.9, 110, 79, 59.6, 59.4, 54.1, 40, 28.5, 27.8, 23, 18.3. HRMS (ESI-Q-TOF): *m*/*z* [M + H]^+^ calcd for C_26_H_33_FN_4_O_2_: 453.2660; found: 453.2651.

- ***tert*-Butyl(4-(((6-bromoisoquinolin-1-yl)methyl)((3-methylpyridin-2-yl)methyl) amino)butyl) carbamate**

This compound was prepared from 1-(chloromethyl)-6-bromoisoquinoline **15d** (100 mg, 0.39 mmol) and *tert*-butyl (4-(((3-methylpyridin-2-yl)methyl)amino)butyl)carbamate **23** (76.25 mg, 0.26 mmol). The title compound was isolated as a brown viscous oil (104 mg, 78%). **^1^H NMR** (400 MHz, CDCl_3_) δ: 8.43 (d, *J* = 5.8 Hz, 1H), 8.40 (d, *J* = 3.9 Hz, 1H), 7.93 (d, *J* = 1.9 Hz, 1H), 7.85 (d, *J* = 9 Hz, 1H), 7.50–7.41 (m, 3H), 7.12 (dd, *J* = 7.6, 4.8 Hz, 1H), 4.65 (s, 1H), 4.15 (s, 2H), 3.83 (s, 2H), 2.96–2.87 (m, 2H), 2.56 (t, *J* = 7.3 Hz, 2H), 2.16 (s, 3H), 1.75–1.60 (m, 2H), 1.58–1.47 (m, 2H), 1.42 (s, 9H). **^13^C NMR** (101 MHz, CDCl_3_) δ: 157.1, 156.4, 146.7, 142.8, 138.4, 137.9, 133.7, 130.4, 129.3, 128.7, 126.5, 125, 123, 119.9, 79.3, 59.7, 54.5, 40.3, 28.8, 28.1, 23.3, 18.7. HRMS (ESI-Q-TOF): *m*/*z* [M + H]^+^ calcd for C_26_H_33_BrN_4_O_2_: 513.1860; found: 513.1854.

- ***tert*-Butyl(4-((isoquinolin-1-ylmethyl)((3-methylpyridin-2-yl)methyl)amino)butyl) carbamate**

This compound was prepared from 1-(bromomethyl)isoquinoline (100 mg, 0.45 mmol) and *tert*-butyl (4-(((3-methylpyridin-2-yl)methyl)amino)butyl)carbamate **15** (88 mg, 0.3 mmol). The title compound was isolated as a brown viscous oil (110 mg, 84%). **^1^H NMR** (400 MHz, CDCl_3_) δ: 8.37 (dd, *J* = 4.7, 1.1 Hz, 1H), 8.05 (d, *J* = 8.4 Hz, 2H), 7.77 (dd, *J* = 8.2, 1.3 Hz, 1H), 7.71–7.65 (m, 1H), 7.54–7.47 (m, 2H), 7.39 (dd, *J* = 7.7, 0.7 Hz, 1H), 7.06 (dd, *J* = 7.6, 4.8 Hz, 1H), 4.74 (s, 1H), 3.91 (s, 2H), 3.86 (s, 2H), 3.06–2.93 (m, 2H), 2.57 (t, *J* = 7.2 Hz, 2H), 2.33 (s, 3H), 1.70–1.61 (m, 2H), 1.59–1.50 (m, 2H), 1.42 (s, 9H). **^13^C NMR** (101 MHz, CDCl_3_) δ: 161.2, 157.5, 156.4, 147.9, 146.6, 138.4, 136.4, 133.6, 129.7, 129.5, 127.9, 127.7, 126.5, 122.8, 121.9, 61.8, 60.1, 54.7, 40.6, 28.9, 28.2, 24.1, 19. HRMS (ESI-Q-TOF): *m*/*z* [M + H]^+^ calcd for C_26_H_34_N_4_O_2_: 435.2754; found: 435.2757.

#### 3.2.11. General Procedure for Deprotection of the Basic *n*-Butylamine Side Chain from Boc-Protected Intermediates 

**General procedure**

To a solution of the Boc-protected intermediate (0.1 mmol) in dry dichloromethane (1 mL) was added trifluoroacetic acid (0.3 mL). The reaction mixture was stirred for 1 h at room temperature. The solvents were evaporated in vacuo. The residue was neutralized by adding a saturated aqueous NaHCO_3_ solution and the aqueous layer was extracted five times with dichloromethane. The combined organic layers were dried over MgSO_4_ and evaporated. The crude residue was purified via silica gel flash column chromatography using a mixture of DCM/MeOH (in a ratio gradually ranging from 95:5 to 90:10) as the mobile phase. The following compounds were made according to this procedure.

- **N1-((6,7-Dimethoxyisoquinolin-1-yl)methyl)-N1-(5,6,7,8-tetrahydroquinolin-8-yl)butane-1,4-diamine (19a)**

This compound was prepared from its Boc-protected precursor (50 mg, 0.09 mmol). The title compound was isolated as a bright brown viscous oil (47 mg, 95%). **^1^H NMR** (400 MHz, DMSO) δ (ppm): 8.43 (d, *J* = 4.5 Hz, 1H), 8.21 (d, *J* = 5.6 Hz, 1H), 8.17 (s, 1H), 7.55 (d, *J* = 5.4 Hz, 1H), 7.48 (d, *J* = 7.9 Hz, 1H), 7.30 (s, 1H), 7.20–7.10 (m, 1H), 5.32 (s, 1H), 4.29 (dd, *J* = 21.5, 12.0 Hz, 2H), 3.99 (s, 3H), 3.92 (s, 3H), 2.15–1.50 (m, 6H), 1.39 (s, 2H), 1.30–1.09 (m, 4H), 1.07–0.93 (m, *J* = 8.3, 6.1 Hz, 4H). **^13^C NMR** (101 MHz, DMSO) δ: 158.51, 158.20, 157.89, 157.59, 152.18, 139.94, 132.61, 123.25, 121.82, 121.65, 119.06, 118.84, 116.52, 115.85, 112.78, 55.51, 52.06, 49.46, 48.60, 45.67, 43.69, 7.17. HRMS (ESI-Q-TOF): *m*/*z* [M + H]^+^ calcd for C_25_H_32_N_4_O_2_: 421.2598; found: 421.2594.

- **N1-((6-Methoxyisoquinolin-1-yl)methyl)-N1-(5,6,7,8-tetrahydroquinolin-8-yl)butane-1,4-diamine (19b)**

This compound was prepared from its Boc-protected precursor (30 mg, 0.06 mmol). The title compound was isolated as a bright brown viscous oil (22 mg, 92%). **^1^H NMR** (400 MHz, CDCl_3_) δ: 9.99 (s, 2H), 8.54 (d, *J* = 3.7 Hz, 1H), 8.51 (d, *J* = 5.8 Hz, 1H), 8.03 (d, *J* = 9.3 Hz, 1H), 7.51 (d, *J* = 5.8 Hz, 1H), 7.41 (d, *J* = 7.2 Hz, 1H), 7.22 (dd, *J* = 9.3, 2.5 Hz, 1H), 7.12 (dd, *J* = 7.7, 4.8 Hz, 1H), 7.08 (d, *J* = 2.5 Hz, 1H), 4.47 (d, *J* = 15 Hz, 1H), 4.05 (d, *J* = 15 Hz, 2H), 3.07 (s, 3H), 3.01–2.46 (m, 6H), 2.12–1.91 (m, 4H), 1.81–1.54 (m, 4H). **^13^C NMR** (101 MHz, CDCl_3_) δ: 161.1, 156.6, 156, 146.9, 141.6, 139, 138, 135, 125.9, 123, 122.7, 120.9, 120.4, 105.3, 60.2, 55.7, 51.6, 51.3, 39.7, 31.1, 29.3, 27.7, 26.9, 21.7, 21.12. HRMS (ESI-Q-TOF): *m*/*z* [M + H]^+^ calcd for C_24_H_30_N_4_O: 391.2492; found: 391.2487.

- **N1-((6-Fluoroisoquinolin-1-yl)methyl)-N1-(5,6,7,8-tetrahydroquinolin-8-yl)butane-1,4-diamine (19c)**

This compound was prepared from its Boc-protected precursor (27 mg, 0.06 mmol). The title compound was isolated as a bright brown viscous oil (21 mg, 99%). **^1^H NMR** (400 MHz, CDCl_3_) δ: 9.84 (s, 2H), 8.60 (d, *J* = 5.8 Hz, 1H), 8.52 (d, *J* = 3.5 Hz, 1H), 8.19 (dd, *J* = 9.3, 5.3 Hz, 1H), 7.57 (d, *J* = 5.8 Hz, 1H), 7.47–7.32 (m, 3H), 7.11 (dd, *J* = 7.6, 4.8 Hz, 1H), 4.46 (d, *J* = 15.1 Hz, 1H), 4.11 (d, *J* = 14.9 Hz, 2H), 2.98–2.52 (m, 6H), 2.12–1.94 (m, 4H), 1.82–1.58 (m, 4H). **^13^C NMR** (101 MHz, CDCl_3_) δ: 164.5, 162, 157.3, 155.8, 147, 142.2, 138.2, 135, 127.4, 124.5, 122.8, 120.7, 118.3, 111.2, 60.3, 52.2, 51.5, 39.6, 29.8, 29.4, 27.7, 26.6, 21.7, 21.2. HRMS (ESI-Q-TOF): *m*/*z* [M + H]^+^ calcd for C_23_H_27_FN_4_: 379.2292; found: 379.2286.

- **N1-((6-Bromoisoquinolin-1-yl)methyl)-N1-(5,6,7,8-tetrahydroquinolin-8-yl)butane-1,4-diamine (19d)**

This compound was prepared from its Boc-protected precursor (22 mg, 0.04 mmol). The title compound was isolated as a bright brown viscous oil (18 mg, 99%). **^1^H NMR** (600 MHz, CDCl_3_) δ: 8.63 (d, *J* = 5.8 Hz, 1H), 8.54 (d, *J* = 3.7 Hz, 1H), 8.18 (d, *J* = 8.9 Hz, 1H), 8.01 (d, *J* = 1.8 Hz, 1H), 7.68 (dd, *J* = 9, 1.9 Hz, 1H), 7.52 (d, *J* = 5.8 Hz, 1H), 7.41 (d, *J* = 7.2 Hz, 1H), 7.12 (dd, *J* = 7.7, 4.7 Hz, 1H), 4.44 (d, *J* = 14.8 Hz, 1H), 4.12 (d, *J* = 14.9 Hz, 2H), 2.91–2.51 (m, 6H), 2.12–1.84 (m, 4H), 1.74–1.54 (m, 4H). **^13^C NMR** (151 MHz, CDCl_3_) δ: 162.4, 158, 147.3, 14306, 136.8, 134.5, 132, 129.7, 128.4, 127.9, 125.5, 123.3, 121.3, 118.1, 67.7, 59.2, 54, 41.8, 28.5, 26.3, 25.8, 20.5. HRMS (ESI-Q-TOF): *m*/*z* [M + H]^+^ calcd for C_23_H_27_BrN_4_: 439.1492; found: 439.1495.

- **N1-(Isoquinolin-1-ylmethyl)-N1-(5,6,7,8-tetrahydroquinolin-8-yl)butane-1,4-diamine (19e)**

This compound was prepared from its Boc-protected precursor (21 mg, 0.05 mmol). The title compound was isolated as a bright brown viscous oil (16 mg, 99%). **^1^H NMR** (400 MHz, CDCl_3_) δ: 10.04 (s, 2H), 8.61 (d, *J* = 3.7 Hz, 1H), 8.20 (d, *J* = 8.7 Hz, 1H), 8.15 (d, *J* = 8.4 Hz, 1H), 7.79 (d, *J* = 7.7 Hz, 2H), 7.58–7.51 (m, 1H), 7.40 (d, *J* = 7.2 Hz, 1H), 7.33 (d, *J* = 8.4 Hz, 1H), 7.13 (dd, *J* = 7.6, 4.8 Hz, 1H), 4.06 (d, *J* = 14.2 Hz, 2H), 3.92 (d, *J* = 14.2 Hz, 1H), 3.03–2.67 (m, 6H), 2.13–1.89 (m, 4H), 1.76–1.53 (m, 4H). **^13^C NMR** (101 MHz, CDCl_3_) δ: 162.4, 158.3, 147.3, 142.8, 136.8, 136.5, 132, 129.7, 127.5, 127.3, 126.8, 124.3, 121.3, 119.2, 67.7, 59.2, 54, 41.8, 28.5, 26.3, 25.8, 20.5. HRMS (ESI-Q-TOF): *m*/*z* [M + H]^+^ calcd for C_23_H_28_N_4_: 361.2387; found: 361.2376.

- **N1-((6,7-dimethoxyisoquinolin-1-yl)methyl)-N1-((3-methylpyridin-2-yl)methyl)butane-1,4-diamine (24a)**

This compound was prepared from its Boc-protected precursor (17 mg, 0.03 mmol). The title compound was isolated as a bright brown viscous oil (13 mg, 99%). **^1^H NMR** (400 MHz, CDCl_3_) δ: 8.52 (d, *J* = 4Hz, 1H), 8.43 (d, *J* = 5.6 Hz, 1H), 7.47 (d, *J* = 5.6 Hz, 1H), 7.44 (d, *J* = 6.9 Hz, 1H), 7.27 (s, 1H), 7.12 (dd, *J* = 7.7, 4.9 Hz, 1H), 7.07 (s, 1H), 4.20 (s, 2H), 4.02 (s, 3H), 4.00 (s, 3H), 3.83 (s, 2H), 3.15 (t, *J* = 5.4 Hz, 2H), 2.69 (t, *J* = 5.3 Hz, 2H), 2.26 (s, 3H), 1.89–1.84 (m, 2H), 1.82–1.77 (m, 2H). **^13^C NMR** (101 MHz, CDCl_3_) δ: 155.3, 153.9, 153, 150.4, 146.7, 140, 138.6, 1336, 131.8, 122.8, 122.6, 119.6, 105.4, 102.3, 57.1, 56.6, 561, 55.2, 39.5, 30.9, 29.6, 27.3, 26.2, 18.5. HRMS (ESI-Q-TOF): *m*/*z* [M + H]^+^ calcd for C_23_H_30_N_4_O_2_: 395.2441; found: 395.2434.

- **N1-((6-Methoxyisoquinolin-1-yl)methyl)-N1-((3-methylpyridin-2-yl)methyl)butane-1,4-diamine (24b)**

This compound was prepared from its Boc-protected precursor (22 mg, 0.05 mmol). The title compound was isolated as a bright brown viscous oil (17 mg, 99%). **^1^H NMR** (400 MHz, CDCl_3_) δ: 8.46 (dd, *J* = 4.8, 1.1 Hz, 1H), 8.41 (d, *J* = 5.8 Hz, 1H), 7.96 (d, *J* = 9.3 Hz, 1H), 7.45 (d, *J* = 5.8 Hz, 1H), 7.41 (dd, *J* = 7.7, 0.7 Hz, 1H), 7.15 (dd, *J* = 9.3, 2.6 Hz, 1H), 7.09 (dd, *J* = 7.6, 4.8 Hz, 1H), 7.03 (d, *J* = 2.5 Hz, 1H), 4.13 (s, 2H), 3.92 (s, 3H), 3.78 (s, 2H), 2.99–2.89 (m, 2H), 2.59 (t, *J* = 5.7 Hz, 2H), 2.20 (s, 3H), 1.76–1.63 (m, 2H). **^13^C NMR** (101 MHz, CDCl_3_) δ: 160.8, 156.9, 155.9, 146.7, 142, 138.7, 132.3, 126.6, 123, 122.7, 120.4, 120.2, 105, 57.7, 57.3, 55.6, 55.2, 39.8, 31.1, 28, 25.6, 18.6. HRMS (ESI-Q-TOF): *m*/*z* [M + H]^+^ calcd for C_22_H_28_N_4_O: 365.2336; found: 365.2330.

- **N1-((6-Fluoroisoquinolin-1-yl)methyl)-N1-((3-methylpyridin-2-yl)methyl)butane-1,4-diamine (24c)**

This compound was prepared from its Boc-protected precursor (28 mg, 0.062 mmol). The title compound was isolated as a bright brown viscous oil (21.5 mg, 99%). **^1^H NMR** (400 MHz, CDCl_3_) δ: 8.51 (d, *J* = 5.8 Hz, 1H), 8.45 (d, *J* = 3.8 Hz, 1H), 8.12 (dd, *J* = 9.3, 5.4 Hz, 1H), 7.52 (d, *J* = 5.8 Hz, 1H), 7.44–7.36 (m, 2H), 7.32 (td, J = 8.9, 2.5 Hz, 1H), 7.08 (dd, *J* = 7.6, 4.8 Hz, 1H), 4.22 (s, 2H), 3.82 (s, 2H), 3.01 (t, *J* = 5.3 Hz, 2H), 2.69 (t, *J* = 5.2 Hz, 2H), 2.22 (s, 3H), 1.86–1.69 (m, 4H). **^13^C NMR** (101 MHz, CDCl_3_) δ: 163.9, 161.4, 158, 156, 146.3, 142.2, 137.9, 132.5, 128.7, 124.5, 122.4, 120.1, 117.1, 110.2, 58.3, 54.7, 40.5, 30.7, 29.5, 24.2, 18.2. HRMS (ESI-Q-TOF): *m*/*z* [M + H]^+^ calcd for C_21_H_25_FN_4_: 353.2136; found: 353.2131.

- **N1-((6-Bromoisoquinolin-1-yl)methyl)-N1-((3-methylpyridin-2-yl)methyl)butane-1,4-diamine (24d)**

This compound was prepared from its Boc-protected precursor (22 mg, 0.043 mmol). The title compound was isolated as a bright brown viscous oil (17.6 mg, 99%). **^1^H NMR** (400 MHz, CDCl_3_) δ: 8.57 (d, *J* = 5.7 Hz, 1H), 8.46 (d, *J* = 3.9 Hz, 1H), 7.97 (d, *J* = 1.8 Hz, 1H), 7.94 (d, *J* = 9.1 Hz, 1H), 7.63 (dd, *J* = 9, 1.9 Hz, 1H), 7.47 (d, *J* = 5.8 Hz, 1H), 7.41 (d, *J* = 7.7 Hz, 1H), 7.09 (dd, *J* = 7.6, 4.8 Hz, 1H), 4.19 (s, 2H), 3.82 (s, 2H), 3.04 (t, *J* = 4.7 Hz, 2H), 2.68 (t, *J* = 5.5 Hz, 2H), 2.22 (s, 3H), 1.87–1.70 (m, 4H). **^13^C NMR** (101 MHz, CDCl_3_) δ: 157.3, 155.2, 146.5, 142.3, 138.4, 137.3, 131.9, 130.8, 129.5, 126.1, 125.4, 125, 122.4, 119.5, 56.9, 55, 39.3, 30.8, 29.5, 27.1, 25.3, 18.4. HRMS (ESI-Q-TOF): *m*/*z* [M + H]^+^ calcd for C_21_H_25_BrN_4_: 413.1336; found: 413.1339.

- **N1-(Isoquinolin-1-ylmethyl)-N1-((3-methylpyridin-2-yl)methyl)butane-1,4-diamine (24e)**

This compound was prepared from its Boc-protected precursor (22 mg, 0.05 mmol). The title compound was isolated as a bright brown viscous oil (17 mg, 99%). **^1^H NMR** (400 MHz, CDCl_3_) δ: 8.43 (dd, *J* = 4.8, 1.1 Hz, 1H), 8.08 (dd, *J* = 8.3, 4.7 Hz, 2H), 7.77 (dd, *J* = 8.1, 1.1 Hz, 1H), 7.74–7.68 (m, 1H), 7.54–7.49 (m, 1H), 7.47 (d, *J* = 8.5 Hz, 1H), 7.41 (dd, *J* = 7.6, 0.9 Hz, 1H), 7.08 (dd, *J* = 7.6, 4.8 Hz, 1H), 3.92 (s, 2H), 3.85 (s, 2H), 2.75 (t, *J* = 6.5 Hz, 2H), 2.58 (t, *J* = 6.8 Hz, 2H), 2.35 (s, 3H), 1.65–1.56 (m, 2H), 1.53–1.43 (m, 2H). **^13^C NMR** (101 MHz, CDCl_3_) δ: 158.1, 155.1, 147.2, 146.4, 138.4, 137.1, 131.7, 130.2, 128.2, 127.4, 127.1, 126.5, 122.4, 122.3, 61, 56.9, 55, 39.2, 30.7, 29.5, 26.9, 25.6, 18.3. HRMS (ESI-Q-TOF): *m*/*z* [M + H]^+^ calcd for C_21_H_26_N_4_: 335.2230; found: 335.2228.

#### 3.2.12. *tert*-Butyl (4-((pyridin-2-ylmethyl)(5,6,7,8-tetrahydroquinolin-8-yl)amino)butyl)carbamate

Picolinaldehyde (1 eq., 0.6887 mmol, 0.0655 mL) was added to a slurry of NaBH(OAc)_3_ (1.78 eq., 1.2259 mmol, 259.8 mg) in dichloromethane (3 mL), followed by the addition of *tert*-butyl (4-((5,6,7,8-tetrahydroquinolin-8-yl)amino)butyl)carbamate **18** (1 eq., 0.6887 mmol, 220 mg). The reaction mixture was stirred at room temperature for 48 h. Then, the reaction was quenched with a 1N NaOH solution to obtain pH ~ 8 of the aqueous layer. The aqueous layer was extracted three times with dichloromethane. The combined organic phases were concentrated and the crude residue was purified via silica gel flash chromatography using a mixture of DCM/MeOH (in a ratio of 96:4) as mobile phase, affording the title compound as a brown viscous oil (177 mg, 63%). **^1^H NMR** (400 MHz, CDCl_3_) δ: 8.49 (d, *J* = 3.7 Hz, 1H), 8.45 (d, *J* = 4.8 Hz, 1H), 7.73 (d, *J* = 7.9 Hz, 1H), 7.63, (td, *J* = 7.5, 1.7 Hz, 1H), 7.33 (d, *J* = 7.5 Hz, 1H), 7.10 (dd, *J* = 6.5, 5.6 Hz, 1H), 7.04 (dd, *J* = 7.6, 4.7 Hz, 1H), 4.8 (s, 1H), 4.13 (d, *J* = 7.4 Hz, 1H), 3.91 (d, *J* = 14.6 Hz, 2H), 3.09–2.94 (m, 2H), 2.86–2.63 (m, 4H), 2.22–2.09 (m, 2H), 2.06–1.95 (m, 2H), 1.94–1.81 (m, 2H), 1.76–1.61 (m, 2H), 1.41 (s, 9H).**^13^C NMR** (101 MHz, CDCl_3_) δ: 162.4, 157.9, 155.9, 148.7, 147.3, 139.7, 136.7, 132, 124.2, 121.2, 121, 79.5, 67.7, 60.9, 54, 35.9, 28.4, 27.7, 25.8, 20.5. HRMS (ESI-Q-TOF): *m*/*z* [M + H]^+^ calcd for C_24_H_34_N_4_O_2_: 411.2754; found: 411.2757.

#### 3.2.13. *tert*-Butyl (4-(((3-methylpyridin-2-yl)methyl)(pyridin-2-ylmethyl)amino)butyl)carbamate 

Picolinaldehyde (1 eq., 1.02 mmol, 0.1 mL) was added to a slurry of NaBH(OAc)_3_ (1,78 eq., 1.82 mmol, 385.7 mg) in DCM (3 mL), followed by the addition of *tert*-butyl (4-(((3-methylpyridin-2yl)methyl)amino)butyl)carbamate **23** (1 eq., 1.02 mmol, 200 mg). The reaction was stirred at room temperature for 48 h. After completion, the reaction mixture was quenched utilizing 1 N NaOH solution to obtain pH ~ 8 in the aqueous layer. After extracting the mixture with DCM for 3 times, the combined organic phases were concentrated. Thereafter, the residue was purified through column chromatography using a DCM/MeOH (96:4) gradient elution to afford the corresponding compound. Brown viscous oil; yield: 206 mg (52%).**^1^H NMR** (400 MHz, CDCl_3_) δ: 8.50 (d, *J* = 4.1 Hz, 1H), 8.36 (dd, *J* = 4.8, 1.2 Hz, 1H), 7.59 (td, *J* = 7.7, 1.8 Hz, 1H), 7.39 (d, *J* = 6.7 Hz, 1H), 7.34 (d, *J* = 10.3 Hz, 1H), 7.12 (dddd, *J* = 7.4, 5, 1.1 Hz, 1H), 7.07 (dd, *J* = 7.6, 4.8 Hz, 1H), 4.81 (s, 1H), 3.80 (s, 2H), 3.75 (s, 2H), 3.06–2.92 (m, 2H), 2.53 (t, J = 7.2 Hz, 2H), 2.30 (s, 3H), 1.57–1.47 (m, 2H), 1.43 (s, 9H), 1.41–1.31 (m, 2H). **^13^C NMR** (101 MHz, CDCl_3_) δ: 160.1, 157.3, 156.4, 149.1, 146.4, 138.3, 136.5, 133.5, 123.7, 122.7, 122.1, 60.7, 59.9, 54.4, 40.4, 28.8, 28.1, 24, 18.7. HRMS (ESI-Q-TOF): *m*/*z* [M + H]^+^ calcd for C_22_H_32_N_4_O_2_: 385.2598; found: 385.2591.

#### 3.2.14. N1-(Pyridin-2-ylmethyl)-N1-(5,6,7,8-tetrahydroquinolin-8-yl)butane-1,4-diamine (**20**)

This compound was synthesized starting from its Boc-protected precursor (23 mg, 0.06 mmol), according to the general deprotection procedure, yielding the title compound as a brownish viscous oil (17 mg, 99%). **^1^H NMR** (600 MHz, CDCl_3_) δ: 9.91 (s, 2H), 8.73 (dd, *J* = 4.9, 0.86 Hz, 1H), 8.58 (d, *J* = 3.9 Hz, 1H), 7.68 (td, *J* = 7.7, 1.8 Hz, 1H), 7.41 (d, *J* = 7 Hz, 1H), 7.29–7.21 (m, 2H), 7.15 (dd, *J* = 7.7, 4.8 Hz, 1H), 3.92 (d, *J* = 13.7 Hz, 2H), 3.73 (d, *J* = 13.7 Hz, 1H), 2.99–2.64 (m, 4H), 2.39–2.20 (m, 2H), 2.11–1.92 (m, 2H), 1.75–1.56 (m, 6H). **^13^C NMR** (151 MHz, CDCl_3_) δ: 162.5, 157.9, 148.7, 17.2, 139.8, 136.7, 131.9, 124.2, 121.3, 121, 67.7, 60.9, 53.9, 41.8, 28.4, 26.3, 25.8, 20.5. HRMS (ESI-Q-TOF): *m*/*z* [M + H]^+^ calcd for C_19_H_26_N_4_: 311.2230; found: 311.2226.

#### 3.2.15. N1-((3-Methylpyridin-2-yl)methyl)-N1-(pyridin-2-ylmethyl)butane-1,4-diamine (**25**)

This compound was synthesized starting from its Boc-protected precursor (30 mg, 0.08 mmol), according to the general deprotection procedure, yielding the title compound as a brownish viscous oil (18 mg, 81%). ^1^H NMR (600 MHz, CDCl_3_) δ: 9.36 (s, 2H), 8.66 (d, *J* = 4.5 Hz, 1H), 8.52 (d, *J* = 4.5 Hz, 1H), 7.65 (td, *J* = 7.7, 1.6 Hz, 1H), 7.43 (d, *J* = 7.5 Hz, 1H), 7.21 (d, *J* = 7.4 Hz, 1H), 7.20 (d, *J* = 5.1 Hz, 1H), 7.12 (dd, *J* = 7.5, 4.9 Hz, 1H), 3.77 (s, 2H), 3.73 (s, 2H), 3.13 (t, *J* = 5.4 Hz, 2H), 2.58 (t, *J* = 5.2 Hz, 2H), 2.25 (s, 3H), 1.85–1.79 (m, 2H), 1.78–1.73 (m, 2H). ^13^C NMR (151 MHz, CDCl_3_) δ: 157.4, 1552, 149.8, 146.8, 138.9, 137.4, 132, 124, 123.1, 122.8, 60.4, 57, 55.1, 39.6, 29.8, 27.3, 262, 18.6. HRMS (ESI-Q-TOF): *m*/*z* [M + H]^+^ calcd for C_17_H_24_N_4_: 285.2074; found: 285.2077.

### 3.3. CXCR4 Binding Assay

The CXCL12^AF647^ binding assay with Jurkat cells has been described previously [30]. Briefly, Jurkat cells were resuspended in assay buffer [Hank’s Balanced Salt Solution (HBSS, Thermo Fisher Scientific), 20 mM HEPES buffer, 0.2% bovine serum albumin (Sigma-Aldrich), pH 7.4] at 3 × 10^5^ cells per sample and then treated with various concentrations of the compound at room temperature for 15 min. Afterwards, the cells were incubated with 2.9 nM CXCL12^AF647^ (in assay buffer) at room temperature for 30 min in the dark. Cells were fixed in 1% paraformaldehyde in DPBS and specific CXCL12^AF647^ binding [i.e., mean fluorescence intensity (MFI)] was quantified by flow cytometry (FACSCanto^TM^ II; Becton Dickinson). Data were analyzed with FlowJo^®^ Software. The 50% inhibitory concentration (IC_50_) was calculated for each compound relative to the negative (i.e., autofluorescence of untreated and unlabeled cells) and positive (i.e., untreated cells exposed to CXCL12^AF647^ only) control.

### 3.4. CXCR4 Calcium Mobilization Assay

The calcium mobilization assay has been described in detail previously [31]. U87.CD4.CXCR4 cells (2 × 10^4^ cells per well in DMEM/10% FBS/0.01 M HEPES) were seeded in gelatin-coated (Sigma-Aldrich; 0.1% gelatin in DPBS) black-walled 96-well plates and incubated overnight at 37 °C and 5% CO_2_. The next day, cells were loaded with the fluorescent calcium indicator Fluo-2 acetoxymethyl (AM) ester (4 μM; Abcam) and incubated at room temperature in the dark for 45 min. Then, cells were incubated with various concentrations of the compounds for 10 min prior to the addition of 6.25 nM CXCL12 (in assay buffer). Fluctuations in intracellular calcium levels were measured in real time by the FLIPR Tetra^®^ (Molecular Devices, Sunnyvale, CA, USA) in all 96 wells simultaneously. The response over baseline (after CXCL12 addition) was calculated with the ScreenWorks 4.0^®^ software (Molecular Devices, Version 4.0, www.moleculardevices.com, accessed on 10 January 2015) by dividing the obtained relative light units (RLUs) through the base line measured just before CXCL12 addition. From this the IC_50_ value for each compound was determined taking into account the negative (i.e., untreated cells without CXCL12 stimulation) and positive (i.e., untreated cells with CXCL12 addition) control samples.

### 3.5. Anti-HIV Assays

#### 3.5.1. Cells

The CD4 cell line MT-4 was obtained from the American Type Culture Collection (Rockville, MD, USA) and cultured in RPMI 1640 medium (Gibco BRL, Gaithersburg, MD, USA) with 10% heat-inactivated fetal calf serum (Biowhittaker Europe, Verviers, Belgium) and 2 mmol/L L-glutamine (Gibco BRL).

#### 3.5.2. Viruses

The HIV-1 molecular clone NL4.3 and the HIV-2 ROD strain were obtained from the NIAID AIDS Reagent Program (National Institutes of Health, Bethesda, MD, USA).

#### 3.5.3. Assay

The anti-HIV-1 and anti-HIV-2 activity in MT-4 cells was determined using a tetrazolium-based colorimetric assay. This assay been described in detail before [29,32]. Threefold dilutions of the drugs in 100 μL medium were added to duplicate wells of 96-well flat bottom plates (Iwaki Glass). Then, MT-4 cells were seeded in the tissue culture plates (7.5 × 10^4^ cells in 50 μL medium), and finally 50 μL diluted HIV-1 NL4.3 and HIV-2 ROD stock (20× the median tissue culture infective dose) was added to each well, resulting in a final volume of 200 μL. The cytopathic effect induced by the virus was checked regularly microscopically. After 4 d of infection, when a strong cytopathic effect was observed in the positive control (i.e., untreated, HIV-infected cells), the cell viability was assessed spectrophotometrically via the in situ reduction of the tetrazolium compound 3-(4,5-dimethylthiazol-2-yl)-5-(3-carboxymethoxyphenyl)-2-(4-sulfophenyl)-2H-tetrazolium inner salt, using the CellTiter 96 AQueous One Solution Cell Proliferation Assay (Promega, Fitchburg, WI, USA). The absorbance was then recorded at 490 nm with a 96-well plate reader and compared with four cell control replicates (cells without virus and drugs) and four virus control wells (cells with virus but without drug). Each assay was performed at least three times. The median inhibitory concentration (EC_50_), or the concentration that inhibited HIV-induced cell death by 50%, was calculated from each dose–response curve. Absorbance was recorded using the VersaMax ELISA™ microplate reader (Molecular Devices) and analyzed with the Softmax Pro^®^ software (Molecular Devices, Version 4.0, www.moleculardevices.com, accessed on 10 January 2015). Using mock-infected cells, the cytotoxic concentration 50 (CC_50_) of each compound was investigated.

## 4. Conclusions

Based on a previously established methodology for the synthesis of 1-methylene-isoquinolines, a new series of isoquinoline-based CXCR4 antagonists was prepared. Besides the synthesis of the unsubstituted isoquinoline congener, also various substituents (monomethoxy, dimethoxy, fluorine, bromine) were introduced on the isoquinoline ring. The head group was either the classical tetrahydroisoquinoline moiety or a 3-methylpyridinyl group. The majority of the compounds do show potent activity in the various assays. All tetrahydroisoquinoline-based derivatives (compounds **19a**–**e** and **20**) were evaluated as racemic mixtures. As it is well-known that in this compound class, the (*S*)-enantiomer is more potent than the (*R*)-enantiomer. Future chemistry will focus on the enantioselective synthesis of the eutomer. Among this new series of analogues, compound **24c** looks very promising, because of its potent CXCR4 antagonism in the binding, as well as in the calcium mobilization assay and its strong and selective anti-HIV activity. Moreover, compound **24c** lacks a chiral center, avoiding the need for any costly enantioselective synthesis.

## Figures and Tables

**Table 1:** CXCR4 and HIV data of final compounds.

	CXCR4		HIV		
Cmpd	Binding IC_50_ (µM) ^a^	Calcium IC_50_ (µM) ^b^	HIV-1 NL4.3 EC_50_ (µM) ^c^	HIV-2 ROD EC_50_ (µM) ^c^	CC_50_ (µM) ^d^
**19a**	0.0072 ± 0.0032	0.025 ± 0.0081	0.074 ± 0.027	0.042 ± 0.013	>100
**19b**	0.0025 ± 0.0012	0.028 ± 0.0049	0.061 ± 0.031	0.077 ± 0.019	11.11 ± 0.54
**19c**	0.0089 ± 0.0056	0.033 ± 0.021	0.016 ± 0.012	0.011 ± 0.0056	18.12 ± 6.82
**19d**	0.026 ± 0.0017	0.44 ± 0.21	0.55 ± 0.36	0.55 ± 0.36	43.58 ± 1.77
**19e**	0.0092 ± 0.0042	0.13 ± 0.041	0.084 ± 0.015	0.026 ± 0.030	26.7 ± 18.61
**20**	0.0026 ± 0.0012	0.057 ± 0.021	0.058 ± 0.071	0.058 ± 0.022	55.45 ± 0.72
**24a**	0.0032 ± 0.00099	0.013 ± 0.0053	0.023 ± 0.008	0.024 ± 0.0018	>100
**24b**	0.89 ± 0.46	3.85 ± 0.19	>9.27	>9.27	9.27 ± 0.46
**24c**	0.00064 ± 0.00017	0.0061 ± 0.001	0.0062 ± 0.0059	0.0038 ± 0.00029	30.77 ± 0.88
**24d**	0.039 ± 0.024	0.69 ± 0.23	2.54 ± 0.91	2.54 ± 0.91	29.48 ± 9.76
**24e**	0.026 ± 0.014	0.93 ± 0.72	0.77 ± 0.29	2.29 ± 0.35	40.89 ± 6.53
**25**	0.0062 ± 0.00057	0.18 ± 0.023	0.23 ± 0.21	0.17 ± 0.16	79.85 ± 13.14
**Plerixafor**	0.025 ± 0.0014	0.26 ± 0.040	0.0049 ± 0.0032	0.0081 ± 0.0042	6.29 ± 0.72

Values are the mean ± SD of at least two independent experiments. ^a^ concentration needed to inhibit CXCL12 receptor binding by 50%; ^b^ concentration needed to inhibit CXCL12-induced calcium signaling by 50%; ^c^ concentration required to achieve 50% protection of MT-4 cells against HIV-induced cytopathicity; ^d^ concentration required to reduce the viability of mock-infected MT4 cells by 50%.

## Data Availability

Not available.

## Sample Availability

Samples of the compounds are available from the authors.
